# Single-Center Clinico-Pathological Case Study of 19 Patients with Cutaneous Adverse Reactions Following COVID-19 Vaccines

**DOI:** 10.3390/dermatopathology8040049

**Published:** 2021-09-27

**Authors:** Dennis Niebel, Joerg Wenzel, Dagmar Wilsmann-Theis, Jana Ziob, Jasmin Wilhelmi, Christine Braegelmann

**Affiliations:** Department of Dermatology and Allergy, University Hospital Bonn, 53127 Bonn, Germany; Joerg.Wenzel@ukbonn.de (J.W.); Dagmar.Wilsmann-Theis@ukbonn.de (D.W.-T.); Jana.Ziob@ukbonn.de (J.Z.); Jasmin.Wilhelmi@ukbonn.de (J.W.); Christine.Braegelmann@ukbonn.de (C.B.)

**Keywords:** COVID-19, adverse event, drug reaction, interface dermatitis, eosinophilia

## Abstract

(1) Background: Coronavirus disease 2019 (COVID-19) vaccines are currently employed on a population-wide scale in most countries worldwide. Data about unusual cutaneous adverse drug reactions (ADR) are scant, though. (2) Methods: We retrospectively analyzed moderate to severe vaccine-related ADR in the Department of Dermatology and Allergy of the University Hospital Bonn between May to June 2021 and analyzed related skin biopsies. (3) Results: As a specialized dermatological academic center, we encountered a total of n = 19 clinically and pathologically heterogeneous cutaneous ADR with a female predominance. Delayed cutaneous ADR occurred as late as 30 days after vaccination. The majority of ADR were mild, though a few patients required systemic treatment (antihistamines, glucocorticosteroids). (4) Conclusions: The clinico-pathological spectrum of cutaneous side effects with COVID-19 vaccines is wide; however, the benefits outweigh the risks by far. More dermatopathological studies on cutaneous ADR not limited to COVID-19 vaccines are desirable to enable a better understanding of underlying pathophysiological mechanisms.

## 1. Introduction

The coronavirus disease 2019 (COVID-19) pandemic struck countries around the globe and resulted in more than 200 million infections and more than 4 million deaths up to August 2021. In light of the severity of the disease, numerous vaccines have been developed rapidly to prevent infection with severe respiratory syndrome coronavirus 2 (SARS-CoV-2) [1]. To enable the return to everyday life without drastic restrictions, the world strives for mass vaccinations to reach immunity on a population-wide scale. Even though the available vaccines display a good tolerability, a few patients experience unusual cutaneous adverse drug reactions (ADR) [2], some of them similar to findings associated with the natural infection [3]. Very early on, there were notable reports of localized erythematous plaques arising late at the site of injection of messenger ribonucleic acid (mRNA) vaccines, which has been labeled “COVID arm” [4]. Moreover, generalized rashes, urticarial reactions, chilblains and many other reactions have been described in recent months [5]. Although the number of reports of rare cutaneous ADR increases by the week, concise dermatohistopathological observations are very limited. Only recently, a retrospective case series with twelve cases was published to identify mixed-cell infiltrates, epidermal spongiosis and interface changes as the most common features associated with COVID-19 vaccine-derived cutaneous ADR [6]. Eosinophils were also a common finding but not always present. One reason for the low number of performed skin biopsies might be that most reactions are mild and wane spontaneously. Within this report, we contribute data of a series of patients who experienced unusual and pronounced cutaneous ADR in the course of different COVID-19 vaccines. We included dermatohistopathological findings whenever available (n = 10). We aimed to highlight the variety of potential cutaneous inflammatory reactions in the course of vaccines to sharpen the focus of physicians who encounter such patients.

## 2. Materials and Methods

### 2.1. Patients

We retrospectively analyzed our medical charts from of May–June 2021 to compile a monocentric case series of moderate to severe vaccine-related cutaneous ADR in the Department of Dermatology and Allergy of University of Bonn, Germany and included a total of n = 19 patients. Patients presented both in the emergency department and in specialized consultation hours (atopic patients, psoriasis, autoimmune skin diseases). Over this period of time, vaccinations in Germany were delivered on a large scale by communal vaccination centers, family doctors and occupational health physicians [7]. Exact numbers about how many people were vaccinated in that given time frame in our metropolitan area are not available; however, at the end of June, more than half of the population of Germany had received at least one dose. At the time of writing, there were four approved vaccines available in our jurisdiction, i.e., BNT162b2, mRNA-1273, AZD1222 and Ad26.COV2.S. With more than 50 million administered doses, BNT162b2 was the most commonly used vaccine in Germany, outnumbering the other three vaccines combined [7]. This explains the composition of the herein mentioned patients with the majority receiving BNT162b2. Notably, patients under the age of 65 who received AZD1222 in the first months of 2021 received an mRNA-based vaccine as the second dose, as advised by the German federal committee on vaccinations (Ständige Impfkommission des Robert Koch Instituts).

### 2.2. Histopathology

Skin biopsies of the most recent lesions were performed, if patients consented. Sections were processed according to standard protocol and stained with hematoxylin–eosin (HE). Further immunohistochemistry was performed if deemed helpful in diagnosis (including specialized stains for T cells, B cells, histiocytes, plasmacytoid dendritic cells and interferon-induced GTP-binding protein Mx1 (MxA), which is an interferon type I/III marker). In suspicion of autoimmune skin diseases, we performed direct immunofluorescence stains in a number of patients (including C3, immunoglobulins G, M and A and fibrinogen) via a frozen section procedure if native material had been obtained during biopsy collection.

## 3. Results

The majority of the patients presenting with cutaneous ADR in the course of COVID-19 vaccines in our department was female (12/19; 63.2%). The mean age of all patients was 48.9 years, but women tended to be younger, with a mean age of 41.9 years as opposed to 60.8 years for male patients. BNT162b2 was the most common trigger, and 15/19 patients (78.9%) had received at least one dose of this agent. The first dose was the cause of the skin eruption more often; however, not all patients decided to receive the second dose after experiencing ADR with the first. Notably, the onset of symptoms ranged from 1 to 30 days, with a mean of 9.4 days. The outcome was very good in almost all patients (Table 1), yet a few patients required immunosuppressive agents such as prednisolone. Less severe reactions could be handled with topical corticosteroids and antihistamines.

Among the ten biopsies studied, interface dermatitis was the most common feature (n = 5). This finding was often accompanied by a patchy lymphocytic inflammatory pattern. Psoriasiform hyperplasia and spongiotic dermatitis were apparent in other cases. Eosinophils were abundant in less than half of the cases (n = 4). In the following, we will describe the most notable cases in more detail.

### 3.1. Delayed Large Local Reaction (“COVID Arm”) with Erythema Nodosum

**Figure 1:**
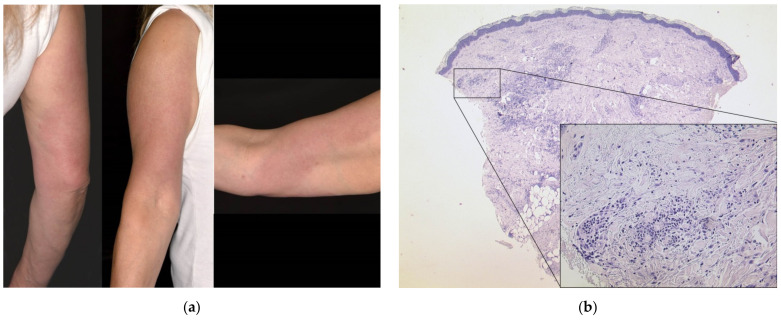
Delayed large local reaction: (**a**) Clinical findings one month after the first dose of BNT162b2: edematous erythema (>10 cm in diameter) on the right upper arm. (**b**) Histological findings of a skin biopsy taken from the right upper arm of the patient showing a superficial and deep lymphocytic infiltration (HE, original magnification 25×; detail HE, original magnification 200×).

A 54-year-old female patient (case #4) experienced erythema and swelling one day after having received the first dose of BNT162b2. She suffered from multiple allergies including house dust mite, gyrase inhibitors and contrast agents. She mentioned sensitization to various cosmetics, which had not been specified further via skin testing. Otherwise, she had no previous dermatological or rheumatologic conditions. Apart from the cutaneous symptoms shortly after the vaccine, she experienced severe musculoskeletal pain and joint stiffness, especially on the affected side (right arm). As the skin reaction persisted over weeks in spite of a therapeutic attempt with prednisolone 10 mg p.o. (Figure 1a), we performed a skin biopsy which displayed a dense superficial and deep perivascular and periadnexial lymphocytic infiltrate with numerous admixed histiocytes and neutrophils (Figure 1b).

**Figure 2:**
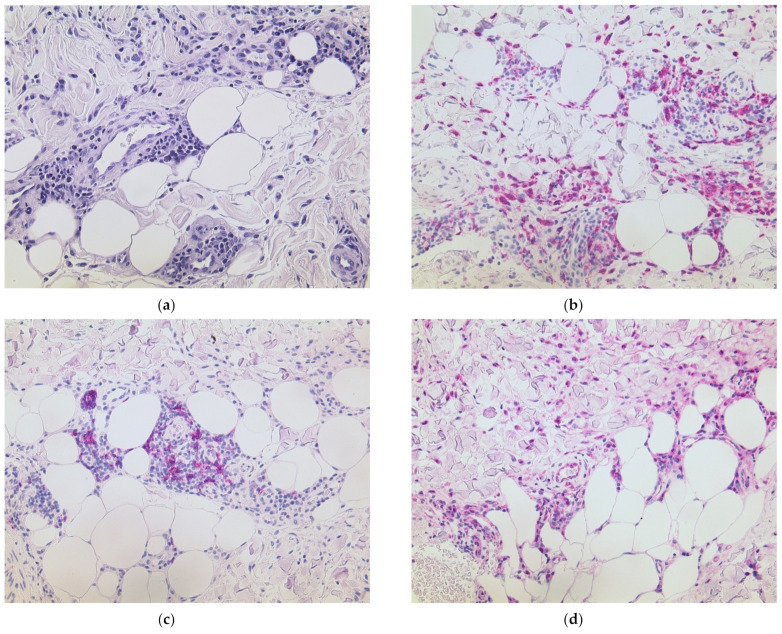
Histological findings of septal panniculitis. (**a**) Mixed inflammatory reaction involving the septae of the subcutaneous fatty tissue (HE, 200× original magnification). (**b**) Abundance of histiocytes (CD68/PGM1, 200× original magnification). (**c**) Abundance of B cells (CD20, 200× original magnification). (**d**) Lesional expression of the type I/III interferon surrogate MxA (MxA, 200× original magnification).

Notably, histology also revealed a septal panniculitis (Figure 2a) which is the hallmark finding of the reactive condition known as erythema nodosum. As the phenomenon of “COVID arm” is not yet exactly understood at present, we aimed to further characterize the inflammatory reaction and performed immunohistochemistry. Apart from a dense T cellular infiltrate, we detected plentiful histiocytes using CD68/PGM1 antibody (Figure 2b) and numerous B cells via CD20 stain (Figure 2c). Furthermore, we identified strong lesional expression of MxA, which correlates with type I/III interferons (Figure 2d). Sarcoidosis could be excluded via further laboratory and radiological workup. The patient required a pulse of prednisolone 1 mg/kg bodyweight to achieve improvement in skin and joint symptoms. The second dose of the same vaccine was administered with a delay of two weeks. This time, she did not experience skin symptoms; however, the joint pain worsened significantly again. A rheumatological re-evaluation resulted in the diagnosis of seronegative rheumatoid arthritis, and the patient now receives methotrexate 15 mg s.c. once weekly and remains under our care.

### 3.2. Generalized Psoriasiform Eruption in an Atopic Patient

**Figure 3:**
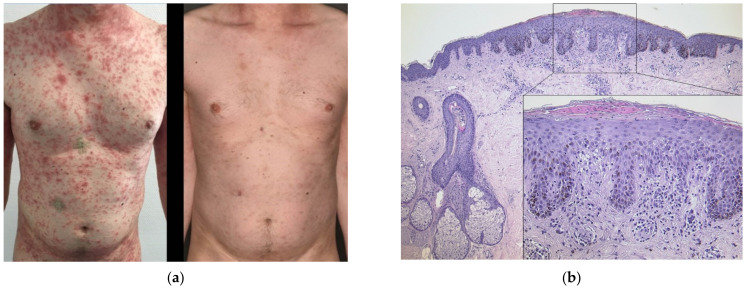
(**a**) Clinical findings five weeks after the first dose of mRNA-based COVID-19 vaccine featuring scaling papules and plaques which had already been present for two weeks (left), and clinical presentation eleven days later with intensified dermatological treatment (right). (**b**) Histological findings of a nevus which was excised to rule out malignancy displaying marked parakeratosis, irregular rete acanthosis and spongiosis, consistent with a psoriasiform eczematous drug reaction (HE, original magnification 50×, detail original magnification 200×). These histological findings were congruent with two other skin biopsies from the trunk.

A 39-year-old male patient (case #5) experienced an impressive pruritic exanthema 21 days after having received the first dose of BNT162b2 (Figure 3a). He had suffered from atopic dermatitis featuring recurrent pruritic eczema of the flexural sides of the extremities for many years. His allergies included various nuts and early-blooming trees. Otherwise, he had no comorbidity. A skin biopsy from the abdomen featured psoriasiform acanthosis, spongiosis and numerous eosinophils; hence, it displayed aspects of both psoriasis and eczema. Interestingly, an excised suspicious nevus also displayed psoriasiform acanthosis with marked parakeratosis (Figure 3b). The patient did not experience improvement with prednisolone 0.5 mg/kg bodyweight per os tapered over a course of five days. Intensified dermatological balneophototherapy involving topical corticosteroids and narrowband UVB311nm light therapy finally led to rapid improvement (Figure 3a). The second dose with the same vaccine was tolerated well without triggering another flare. Scaling and hyperpigmentations remained for weeks, though.

### 3.3. Hematogenous Contact Dermatitis

**Figure 4:**
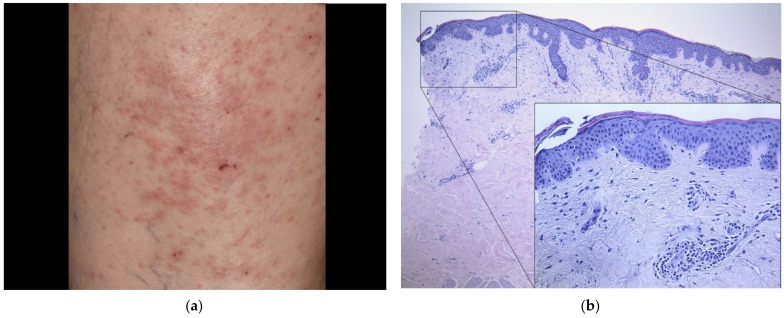
(**a**) Clinical findings four weeks after the second dose of BNT162b2 resembling an eczematous reaction distant from the injection site on the dorsal upper thigh. (**b**) Histological findings of a skin biopsy taken from the trunk of the patient 14 days after the second dose of the vaccine showing parakeratosis and slight dermal edema with neutrophils and eosinophils (HE, original magnification 50×, detail original magnification 200×).

A 77-year-old male patient (case#6) experienced a pruritic eczematous and partly urticarial reaction three days after having received the first dose of BNT162b2. He had an atopic diathesis in terms of allergic rhinitis; known allergies included alder, hazel, beech, birch, sorrel and plantain. Otherwise, he had no significant comorbidity other than Tolosa–Hunt syndrome. He received antihistamines, and, finally, the eruption waned with topical corticosteroids over the next weeks. However, the same affected areas relapsed shortly after the second dose of the same vaccine was administered (Figure 4a). A skin biopsy from the sacrum revealed discrete parakeratosis and slight dermal edema with an admixture of neutrophils and eosinophils compatible with a diagnosis of hematogenous contact dermatitis (Figure 4b). In spite of a protracted course, no systemic medication was necessary to treat the cutaneous ADR.

### 3.4. Flare of Psoriasis

**Figure 5:**
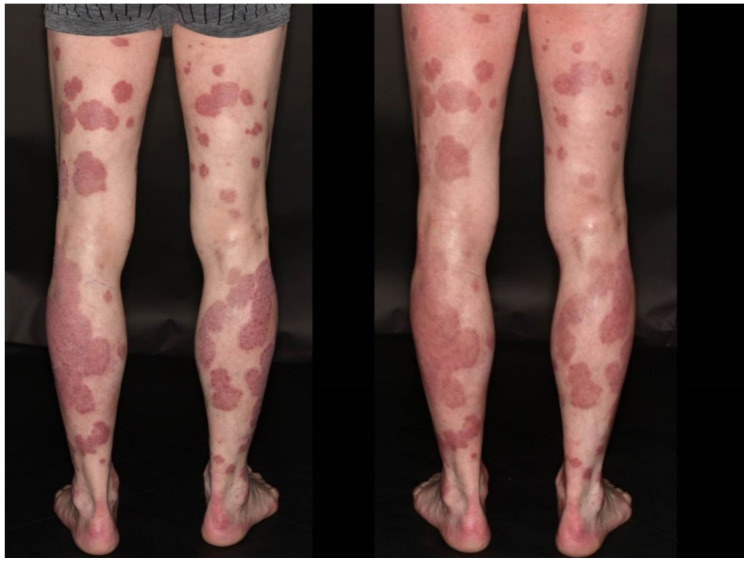
Clinical appearance featuring a persisting exacerbation of scaling erythematous plaques on the lower extremities after having received two doses of BNT162b2 (**left**), and remaining lesions after two weeks of balneophototherapy using dithranole and narrowband ultraviolet B 311nm in our clinic (**right**).

A 62-year-old male patient (case #8) had been diagnosed with psoriasis more than forty years ago and had never required systemic therapy. He gradually experienced worsening of his skin after having received the second dose of BNT162b2. In spite of daily topical treatment with topical corticosteroids, he could not achieve disease remission; hence, we initiated balneophototherapy in our clinic. The psoriasis area and severity index (PASI) was estimated to be 23 four months after full vaccination (Figure 5). His allergies included hazel and grass. He had a medical history of larynx carcinoma in full remission. In light of the severity of psoriasis and persistent lesions in spite of distinguished dermatological treatment in our clinic (Figure 5), we decided to initiate systemic treatment with tildrakizumab, a monoclonal IL23 inhibitor. The patient’s skin has now considerably improved, and the medication with the biologic is continued.

### 3.5. Pityriasis Rosea

**Figure 6:**
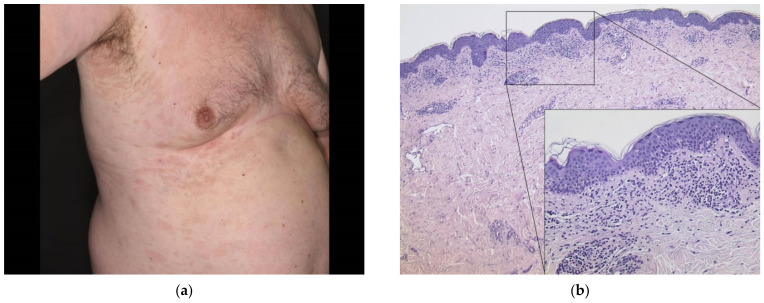
(**a**) Clinical findings three weeks after the first dose of AZD1222 featuring a non-scaling exanthema with distribution following the Langer lines. (**b**) Histological findings of a skin biopsy taken from the trunk of the patient showing a superficial perivascular lymphocytic infiltrate with interface dermatitis and erythrocyte extravasation (HE, original magnification 50×; detail original magnification 200×).

A 63-year-old male patient (case#19) experienced a non-pruritic pale erythematous exanthema of the trunk three weeks after having received the first dose of AZD1222. Singular lesions measured up to 5 cm and were distributed along the Langer lines (“Christmas tree pattern”) (Figure 6a). He had an atopic diathesis in terms of allergic rhinitis; known allergies included cat hair and grass. Otherwise, he had no significant comorbidity other than hypothyroidism. A skin biopsy from the trunk revealed pronounced interface dermatitis and sparse erythrocyte extravasation (Figure 6b) compatible with a diagnosis of pityriasis rosea. We advised watchful waiting with the use of topical corticosteroids on larger lesions and otherwise emollients only. The skin rash waned over the course of weeks. As of the updated recommendations, the pending second vaccine is scheduled with another agent (mRNA vaccine, BNT162b2 in this patient) after three months.

## 4. Discussion

The diverse landscape of cutaneous ADR is an ever-fascinating topic regarding both clinical and histopathological aspects. The variability between localized reactions (e.g., fixed drug eruption), on the one hand, and self-limiting generalized (e.g., maculopapular exanthema) to life-threatening generalized severe cutaneous ADR (e.g., Stevens–Johnson syndrome/toxic epidermal necrolysis), on the other, is astonishing. The pathological variability is not less impressive with reactions involving mainly the epidermis (e.g., symmetric drug-related interflexural exanthema), the epidermo-dermal junction (e.g., drug-induced cutaneous lupus erythematosus), the upper dermis (e.g., drug reaction with eosinophilia and systemic symptoms) or the subcutis (e.g., drug-induced septal panniculitis) [10]. Vaccines may be considered as specific drugs with the purpose to achieve a protective immune response; hence, they bear class-specific side effects [11]. The newly developed COVID-19 vaccines are no exception, and numerous studies have addressed this topic [12,13,14,15,16,17]. We encountered clinically and histologically strikingly different cutaneous adverse reactions in the course of both mRNA-based and viral vector-based COVID-19 vaccines. Some of the described cutaneous ADR appeared later than twenty days after vaccine delivery, and they might be overseen in standard study protocols, accordingly [5,14,15,17]. In the following, we will put our results into perspective with the available literature and outline the current point of view about the occurrence of distinct dermatoses in connection with COVID-19 vaccines.

It is important to recapitulate that vaccines elicit specific immunogenic mechanisms that shift the acquired immune system towards a Th1 phenotype [18]. Hence, it is not surprising that we encountered four patients with clinical and histopathological findings resembling variations of cutaneous lupus erythematosus. Three patients could achieve disease control with a short pulse of prednisolone, which is a meaningful finding. Unfortunately, one previously healthy young woman (case #16) experienced recurrent joint pain and fever and finally required hospitalization. Based on extensive laboratory and radiological examinations, systemic lupus erythematosus and rheumatic arthritis were excluded. Scheduling of the second vaccine dose in case of severe ADR with the first dose is an unsolved problem with these patients. Low-dose prednisolone around the date of the second dose might decrease the susceptibility for another flare of rheumatic conditions [8]. Based on a shift towards Th1 immunity, it is also comprehensive to see diseases resembling paraviral epiphenomena such as pityriasis rosea (PR) or erythema multiforme (EM). As expected, both our encountered PR patients had an excellent course, omitting aggressive treatment (case#13 and case#19). Notably, both mRNA vaccines and viral vector vaccines seem to potentially be causative agents. As with the etiology of PR itself, COVID-19-associated PR and COVID-19 vaccine-associated PR remain largely unexplained phenomena, thus far. The available biopsies showed a rather moderate inflammatory reaction, though the clinical appearance was decisive.

Another condition that typically aggravates with vaccine-derived abundant expression of IFN-γ and TNF-α is psoriasis vulgaris. There are already other case reports about exacerbation of the disease in association with mRNA vaccines [19] similar to case #8. Management should be based on available guidelines as there are numerous excellent treatment options including biologics (anti-IL17, anti-IL23, anti-TNF-α). More dangerously, pustular flares of psoriasis have been described in association with COVID-19 vaccines [20]. We encountered a patient with a succulent erythematous exanthema associated with fever and malaise ten days following mRNA vaccination (case#10). The histology of a skin specimen displayed marked dermal interstitial neutrophilia, and the patient improved markedly with initiation of prednisolone treatment. Although the clinical criteria were not fulfilled, we consider this case as being similar to acute febrile neutrophilic dermatosis (Sweet’s syndrome) which may be associated with infections and vaccination [21]. The skin symptoms waned over the course of a week; unfortunately, the patient experienced continued fatigue, nausea and, later on, hypermenorrhagia. The second dose was adjourned according to the wish of the patient.

Counterintuitively, chronic inflammatory dermatoses including AD may worsen with COVID-19 vaccines although they are mainly Th2-driven [14,22]. Notably, we encountered a patient with long-standing, well-controlled AD who developed a generalized flare with a histologically psoriasiform pattern upon mRNA vaccination (case#5). Balneophototherapy was necessary for disease control in our patient after failure of a short pulse of oral corticosteroids. Curiously, the second dose of the same vaccine was well tolerated, and only scaly skin and slight hyperpigmentation remained over the weeks after the disseminated cutaneous eruption.

Some cutaneous ADR seem to resemble the facultative skin lesions found in some COVID-19 patients [2]. We noted two cases of vesicular reactions (case #7 and 15), but both were mild and resolved quickly with topical anti-inflammatory and antibiotic ointments, respectively. Hence, they were inaccessible for a dermatohistopathological examination. Moreover, acute urticaria and urticarial reactions occur frequently following COVID-19 vaccination, which is also one of the main cutaneous findings in some COVID-19 patients [3]. Patients #11 and #18 were both female and comparably young, had no history of allergy or dermatological comorbidity and could be controlled readily with antihistamines. An anaphylactic reaction could be excluded in the absence of other symptoms. The second dose is pending in one case, and the other woman did not experience another flare which is a meaningful finding underlining the situational context as a cofactor. Antihistamines may be advised liberally in these situations as they are well tolerable and have little side effects.

Components of vaccines such as polysorbates may elicit delayed hypersensitivity in susceptible individuals [23]. The result is acute spongiotic dermatitis that exceeds the spectrum of a “normal” injection site reaction. Generalized dermatitis may be the result of hematogenous spread (compare to cases #6, 12 and 14). All of our patients suffering from eczematous reactions had previously known type IV sensitizations, and two suffered from recurrent eczema in terms of stasis dermatitis or dyshidrotic eczema in the past, which obviously puts them at risk for cutaneous ADR with COVID-19 vaccines. Luckily, all patients could receive full vaccination, and the skin symptoms could be controlled with standard of care treatment. Case #4 is an example of a very large erythematous plaque that persisted weeks after the vaccination. “COVID arm” was first described with the mRNA-1273 vaccine [4,24]. However, similar reactions have now also been published with other COVID-19 vaccines [25]. Histological evaluations of these lesions are scarce. Some authors described a superficial and deep perivascular lymphocytic infiltrate with dilated vessels and intraluminal neutrophils [16], while others found admixed eosinophils, which are typically involved in hypersensitivity reactions [26]. An involvement of the subcutis in terms of septal panniculitis (erythema nodosum) is a finding which has not been reported previously to the best of our knowledge. Admittedly, a case report described peculiar skin lesions resembling erythema nodosum upon infection with SARS-CoV-2 [27]. Further histological reappraisal of the underlying immune reaction is needed which would be a useful implementation into future clinical trials.

The limitations of our study include the randomness of patient referral to our center, missing validity to incidences of specific reactions and the predominance of mRNA-based vaccines in our patient population. Larger studies are needed to better comprehend the variability of drug and vaccine reactions employing variability of ethnicity, sex and environmental or situational factors. Ultimately, the goal is to avoid as many ADR as possible and to allow safe mass vaccinations in heterogeneous populations.

## 5. Conclusions

COVID-19 vaccines often lead to self-limiting cutaneous ADR including erythema and swelling at the site of injection, yet they may elicit heterogeneous delayed cutaneous ADR. The clinical and histopathological spectrum is very broad; thus far, the available data are limited and do not allow reliable predictions about which patient groups are at greatest risk. The incidence of severe reactions appears to be very low; therefore, vaccines should be generously offered to the population to prevent further spread of SARS-CoV-2.

## Figures and Tables

**Table 1:** Tabular overview of cutaneous adverse vaccine reactions of the Department of Dermatology and Allergy of the University Hospital Bonn from May until June 2021. Abbreviations: F—female; M—male; NA—not available; p.o.—per os; s.c.—subcutaneous; CLE—cutaneous lupus erythematosus; MCTD—mixed connective tissue disease; AD—atopic dermatitis; TCS—topical corticosteroids; UVB311nm—narrowband ultraviolet B light therapy.

#	Sex	Age	Dose	Vaccine	Onset	Clinical	Histological	Comorbidity	Diagnosis	Management	Outcome
1	F	73	1	BNT162b2	10 d	Ill-defined erythematous plaques on the trunk	NA	CLE	Flare of CLE	Prednisolone 1 mg/kg tapered over three weeks	Excellent; no flare with second dose BNT162b2 [8]
2	M	41	1	BNT162b2	5 d	Generalized erythematous annular plaques	Patchy lymphocytic infiltrate, slight interface dermatitis	MCTD	Drug-induced CLE	Prednisolone 1 mg/kg tapered over three weeks; continuation of hydroxychloroquine 200 mg 2 x daily; methotrexate 15 mg s.c.	Excellent, no flare with second dose [9]
3	F	50	1	BNT162b2	30 d	Periorbital erythema and edema, V-sign	NA	None	Dermatomyositis	Prednisolone 1 mg/kg tapered over six weeks	No flare with second dose; diagnostic workup to exclude malignancy pending
4	F	54	1	BNT162b2	1 d	Upper arm and shoulder erythematous and edematous, forearm swollen	Dense lymphocytic infiltrate with numerous histiocytes and neutrophils, septal panniculitis	None	“COVID arm”, protracted development of erythema nodosum	Prednisolone 1 mg/kg tapered over three weeks	Second dose with BNT162b2 two weeks delayed; skin improved, protracted course of arthritic pain → diagnosis of rheumatoid arthritis
5	M	39	1	BNT162b2	21 d	Generalized eczematous plaques	Psoriasiform acanthosis, spongiosis, eosinophilia	Atopic diathesis	Psoriasiform flare of AD	Prednisolone 0.5 mg/kg tapered over a week; TCS 2 x daily, UVB311nm	Excellent; no flare with second dose
6	M	77	1	BNT162b2	3 d	Localized eczematous and urticarial plaques	Parakeratosis, dermal edema with eosinophils, interface dermatitis	Atopic diathesis	Hematogenous contact dermatitis	TCS 2 x daily	Protracted course with flare after second dose
7	F	55	2	BNT162b2	7 d	Grouped pruritic papulovesicles	NA	Atopic diathesis, chronic spontaneous urticaria	Vesicular reaction	Topical fusidine ointment 2 x daily	Excellent
8	M	62	2	BNT162b2	20 d	Generalized erythemato-squamous plaques	NA	Psoriasis vulgaris	Flare of psoriasis	Cignoline, TCS 2 x daily, UVB311nm, tildrakizumab	Excellent
9	M	76	2	BNT162b2	2 d	Petechial annular plaques on the lower extremities	NA	IgG/IgM cutaneous immune complex vasculitis	Flare of immune complex vasculitis	Continued use of dapsone, prednisolone 10 mg p.o. for a week, 5 mg p.o. maintenance	Excellent
10	F	30	1	BNT162b2	10 d	Generalized non-scaling erythematous plaques	Edematous papillary dermis with admixed neutrophils	None	Neutrophilic drug eruption	Prednisolone 1 mg/kg tapered over six weeks	Protracted course with fatigue and nausea; second dose adjourned
11	F	15	1	BNT162b2	2 d	Generalized hives	NA	None	Acute urticaria	Antihistamines	Second dose scheduled with BNT162b2 after six weeks, no flare with second dose
12	M	68	1	BNT162b2	2 d	Scaling erythematous plaques on extremities	Spongiotic dermatitis, dermal edema with eosinophils	Stasis dermatitis	Hematogenous contact dermatitis	TCS 2 x daily	Excellent; second dose adjourned (wish of patient), allergological diagnostics anticipated
13	F	40	2	BNT162b2	8 d	Generalized non-pruritic scaling plaques	NA	None	Pityriasis rosea	TCS 2 x daily	Excellent
14	F	67	2	BNT162b2	12 d	Pruritic scaling erythema in light-exposed areas	Parakeratosis, spongiosis, eosinophils and neutrophils	Chronic hand eczema	Hematogenous contact dermatitis with flare of chronic hand eczema	Prednisolone 10 mg p.o. for a week, ciclosporine raised from 50 to 100 mg p.o., TCS 2 x daily	Steady improvement over weeks, chronic lesions persistent
15	F	49	2	AZD1222/BNT162b2	3 d	Grouped pruritic papulovesicles	NA	None	Vesicular reaction	TCS 2 x daily	Excellent
16	F	22	1	mRNA-1273	10 d	Elevated erythematous annular papules and plaques without scaling	Superficial and deep lymphocytic infiltrate with interface dermatitis	None	Drug-induced CLE	Prednisolone 1 mg/kg tapered over three weeks; etoricoxib 90 mg p.o.	Recurrent joint pain and fever, hospitalization, second dose adjourned [9]
17	F	20	2	mRNA-1273	9 d	Generalized pruritic exanthema	Superficial and deep lymphocytic infiltrate with interface dermatitis	None	Drug-induced CLE	Prednisolone 10 mg p.o., antihistamines, TCS 2 x daily	Excellent
18	F	38	1	AZD1222	1 d	Generalized hives	NA	None	Acute urticaria	Antihistamines	Second dose scheduled with BNT162b2 after three months (pending)
19	M	63	1	AZD1222	22 d	Pale erythematous maculae along Langer lines	Interface dermatitis and erythrocyte extravasation	Atopic diathesis	Pityriasis rosea	TCS on demand, emollients, “watchful waiting”	Second dose scheduled with BNT162b2 after three months (pending)

## Data Availability

Data sharing is not applicable to this article as no datasets were generated or analyzed during the current study.
